# Differential Effect of Light and Dark Period Sleep Fragmentation on Composition of Gut Microbiome and Inflammation in Mice

**DOI:** 10.3390/life11121283

**Published:** 2021-11-23

**Authors:** Larry D. Sanford, Laurie L. Wellman, Richard P. Ciavarra, Edward C. Oldfield, Rouzbeh Shams, Jennifer L. Copare, David A. Johnson

**Affiliations:** 1 Sleep Research Laboratory, Center for Integrative Neuroscience and Inflammatory Diseases, Pathology and Anatomy, Eastern Virginia Medical School, Norfolk, VA 23507, USA; Wellmall@evms.edu; 2 Microbiology and Molecular Cell Biology, Eastern Virginia Medical School, Norfolk, VA 23507, USA; ciavarrp@evms.edu; 3 Gastroenterology Division, Department of Internal Medicine, Eastern Virginia Medical School, Norfolk, VA 23507, USA; ecoldfield@gmail.com (E.C.O.IV); rouzbehshams@gmail.com (R.S.); jencopare@gmail.com (J.L.C.); dajevms@aol.com (D.A.J.)

**Keywords:** chemokine, cytokine, immune system, microbiome, neuroinflammation

## Abstract

Bi-directional interactions amongst the gut microbiota, immune system, and brain function are thought to be critical mediators of health and disease. The role sleep plays in mediating these interactions is not known. We assessed the effects of sleep fragmentation (SF) on the microbiota–gut–brain axis. Male C57BL/6NCrl mice (4 to 5 per cage, fed standard lab chow) experienced SF via mechanical stimulation at 2 min intervals during the light (SF) and dark (DD, dark disturbances) periods. Home cage (HC) controls were undisturbed. After 10 days, fecal samples were collected at light onset, midday, light offset, and midnight. Samples were also collected after 10 days without SF. Subsequently, the mice were randomized across groups and allowed 20 additional days of recovery followed by 10 days of SF or DD. To assess effects on the microbiota, 16S rRNA sequencing was used, and mesenteric lymph nodes (MLNs) and cortex and medial prefrontal cortex were analyzed using cytokine arrays. SF and DD produced significant alterations in the microbiota compared to HC, and DD had greater impact than SF on some organisms. SF produced marked suppression in MLNs of chemokines that regulate inflammation (CCL3, CCL4 and their receptor CCR5) and maintain the immune mucosal barrier (Cxcl13) at the same time that cortical cytokines (IL-33) indicated neuroinflammation. DD effects on immune responses were similar to HC. These data suggest that SF alters the microbiome and suppresses mucosal immunity at the same time that mediators of brain inflammation are upregulated. The translational implications for potential application to clinical care are compelling.

## 1. Introduction

Studies have strongly implicated the various microorganisms that inhabit and share our bodies in mediating health and disease and behavior via influences on the immune system and brain. Influences are believed to be bi-directional. For example, while gut microbiota may modulate brain function, the brain may alter gut microbiota by changing gastrointestinal motility and intestinal permeability [1,2]. There is also a potential relationship between sleep disturbances and gut microbiota that appear to have relevance for disease [3,4,5,6]. However, the effects of sleep disruption are complex as it also has a significant impact on the immune system [7,8,9,10,11] that would have the potential to alter the effects of the gut microbiota, possibly altering the immune processes that maintain the barrier between host tissue and the microbiota and/or functioning of the central nervous system. An extreme example of this is found in animals experiencing prolonged, experimental sleep deprivation. Sustained sleep deprivation promotes bacterial translocation [12], and death that can occur after prolonged sleep deprivation in animals has been attributed to bloodstream infection by opportunistic anaerobic bacteria that normally are contained within the gut [13]. Thus, while experimental prolonged sleep deprivation does not model sleep disturbances common in modern life, the recognized effects of sleep deficiency and increasing recognition of the role that the gut microbiota play in mediating immune function suggest that delineating the interactions among disturbed sleep, the immune system and gut microbiota was critical for understanding their role in health and disease.

In this study, we examined the effects of disturbed sleep on elements of the microbiota–gut–brain (M–G–B) axis using a mouse model of sleep fragmentation (SF). SF disrupts the continuity of sleep and interferes with its restorative effects [7] without necessarily significantly altering total sleep time. SF is highly prevalent in modern life as it can arise from stress [14], increases with aging [15] and it is a hallmark of sleep apnea [15], a condition linked to obesity and that has been associated with a variety of health risks [15]. Critically, SF is significant because it can arise from a variety of environmental- (e.g., stress-related), physiological- (e.g., aging) and disease-related (e.g., sleep apnea) causes that have the potential to impact health. It also has been reported to increase food intake, increase growth in Lachnospiraceae and Ruminococcaceae and decrease Lactobacillaceae families, produce systemic and visceral white adipose tissue inflammation, and to alter insulin sensitivity [16]. 

We examined expression of chemokines/cytokines in the mesenteric lymph nodes (MLNs) as measures of the effects on the mucosal immune response and in selected brain regions (cortex, medial prefrontal cortex (regulates stress response)) as measures of the neuroimmune response using qPCR inflammatory cytokine arrays. We also compared the effects of disruptions conducted in the light period (major sleep period of rodents) and in the dark period (when they are more active) to assess effects specific to disrupted sleep relative to those produced by forced movement during the wake period. 

## 2. Materials and Methods

### 2.1. Subjects

Male C57BL/6NCrl (B6) mice were obtained from Charles River (Wilmington, MA, USA). The mice were 8–9 weeks old and weighed 20–25 g at arrival. The animals were group housed and were kept in a colony room with food (Envigo, 2014 Teklad global 14% protein rodent maintenance diet) and water available, ad libitum. The colony room was maintained on a 12:12 light–dark cycle and ambient temperature at 24 °C ± 1.5 °C. At least one week for acclimation was allowed before the experiment was started. Throughout the experimental procedures, measures were taken to minimize unnecessary pain and discomfort of the animals. All procedures were conducted in accordance with the National Institutes of Health Guide for the Care and Use of Experimental Animals and were approved by Eastern Virginia Medical School’s Institutional Animal Care and Use Committee (protocol 17-015, approved 15 September 2017).

### 2.2. Procedures

#### 2.2.1. Sleep Fragmentation and Collection of Fecal Samples

SF was performed using a commercial, validated device (Lafayette Instruments, Sleep Fragmentation Chamber, model 80391) that employs an automated sweeper arm that moves across animal cages to disrupt sleep via tactile stimulation [7,17]. This SF protocol (2 min between each sweep) reportedly produces moderate to severe SF [7,8,17] without significantly reducing overall sleep or significantly impacting sleep macro- or micro-architecture [7,17]. Studies have also reported that this procedure is not associated with measurable increases in stress hormones, [7,17] but can impair cognitive performance [7] and can have a negative impact on health including promoting obesity [18] and tumor formation [8]. 

To conduct SF, mice were housed (4–5 per cage) in the SF devices. One group was subjected to SF (2 min intervals between each sweep) during the light period (normal sleep time). Another group received identical treatment during the dark period (DD, dark disturbance group) and another served as a home cage control with no sleep interruption (HC). After 10 days of SF or DD, fecal samples were collected at light onset, midday, light offset, and midnight. Subsequently, the mice were allowed to sleep undisturbed for an additional 10 days and fecal samples were again collected at 6 h intervals. Time matched samples were collected from undisturbed HC mice that never experienced SF or DD.

Fecal samples were processed using MoBio Power Mag kits (MO BIO Laboratories, Inc., Carlsbad, CA, USA). All samples were then cataloged by identifiers containing animal, group and sample period and analyzed using 16S rRNA Sequencing (conducted at the DNA Sciences Core, University of Virginia). Briefly, library preparation and sequencing were conducted on an Illumina MiSeq instrument using the Illumina 16s metagenomics protocol with 15% PhiX spike-in as control. Fastq generation and 16s riboRNA assignments were conducted using Illumina Metagenomics software. Further statistical comparisons and unsupervised clustering classification of the samples were conducted using the DNA-Chip Analyzer (dChip) software package.

#### 2.2.2. Sleep Fragmentation and Tissue Collection

After completion of collecting fecal samples, the mice were randomized across groups and allowed 20 days of recovery followed by 10 additional days of SF or DD prior to sacrifice for assessment of chemokine/cytokine profiles. The mice were euthanized at the end of the last SF session and tissue was collected for analyses of chemokines/cytokines in the mesenteric lymph nodes (MLNs) as measures of the effects on the mucosal immune response and in select brain regions (cortex and medial prefrontal cortex) as measures of the neuroimmune response. RNA was purified from homogenized tissue using the QuickGene RNA tissue kit SII (RT-S2) from FUJIFILM Corporation following the manufacturer’s instructions. Individual RNA samples were pooled and proinflammatory cytokine profiles characterized by real-time PCR using commercial inflammatory cytokine arrays (Qiagen, catalogue number PAMM-0150Z, Hilden, Germany). Cytokine mRNA levels from HC mice were used as controls to calculate fold differences in the experimental groups. Input RNA was normalized using house-keeping genes provided with each array.

### 2.3. Statistics

Comparisons across groups were conducted using between subjects, one-way ANOVAs. Post hoc comparisons among means, when indicated by a significant overall ANOVA, were conducted using Holm–Sidak tests (to maintain error rate at *p* < 0.05). Treatment assignment and sampling periods were coded and blinded to investigators who performed the data preparation, sequencing and subsequent data analyses. The Shannon index was calculated and compared across groups and across treatment conditions and sample periods to assess potential effects of SF on alpha diversity of gut microbiota species.

## 3. Results

### 3.1. SF and DD Produce Different Changes in Gut Microbiota

**Figure 1:**
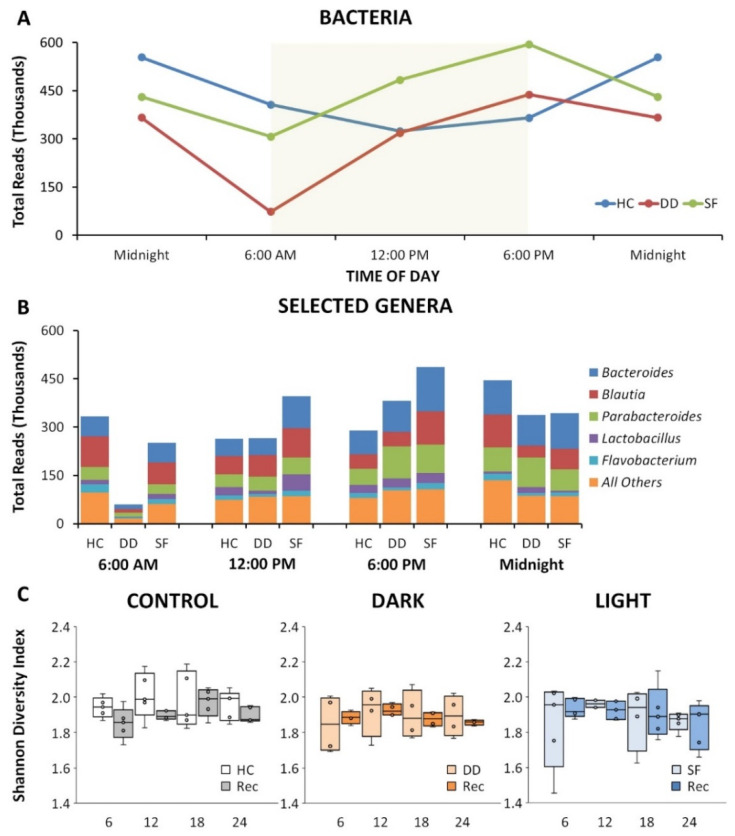
Sleep fragmentation (SF) alters gut microbiota. (**A**) Disruption of circadian microbiome pattern following SF (red) and dark disturbed (DD, blue) mice compared to home cage control (green) mice determined as total bacterial 16S RNA sequencing reads. Yellow box represents lights on in a 12 h ON/12 h OFF light cycle in the animal room. (**B**) Break-down of reads by top represented genera over the 24 h specimen collection time for HC, DD and SF highlighting a compelling suppression of reads following DD and the overall inversion of circadian pattern for SF. (**C**) Box and whisker plots of alpha diversity plotted in 6 h increments for HC, DD and SF groups under sleep fragmentation and recovery conditions. There was no significant change across groups or conditions.

Our studies show that both SF and DD altered the gut microbiome, but had different effects. As depicted in Figure 1A, SF and DD induced alterations in the circadian rhythm for total reads of all genera relative to undisturbed cohorts. Reads for selected genera after 10 days of SF, DD or time matched HC mice are presented in Figure 1B. Differences could vary across groups and with time of day, both light and DD could perturb the microbiota relative to control, and DD had a greater impact than SF on some organisms. Alpha diversity did not differ across groups or across time (Figure 1C).

**Figure 2:**
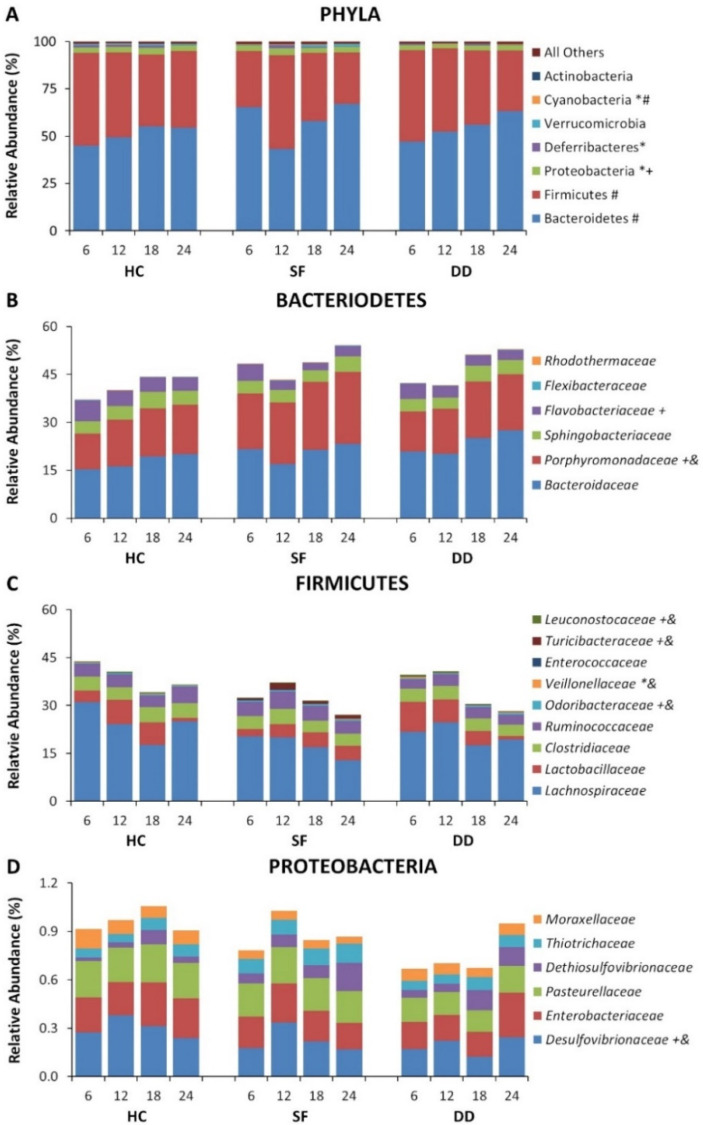
Relative abundance of selected microbiota for the home cage control (HC), dark disturbed (DD) and sleep fragmented (SF) experimental groups plotted at 6 h sampling intervals beginning at light onset. (**A**) Relative abundance at the phylum level. (**B**) Relative abundance at the family level for Bacteroidetes. (**C**) Relative abundance at the family level for Firmicutes. (**D**) Relative abundance at the family level for Proteobacteria. Significance is indicated beside the classification name: *, *p* < 0.05, SF compared to control; +, *p* < 0.05 DD compared to control; &, *p* < 0.05 DD compared to SF; #. *p* < 0.05 across sampling periods (indicated only for Phyla). All Pairwise Multiple Comparison Procedures performed using the Holm–Sidak method. Data at the phylum level are shown as a percentage of all identified phyla. Data at the family level are shown as a percentage of the total identified sequences for the families shown.

**Figure 3:**
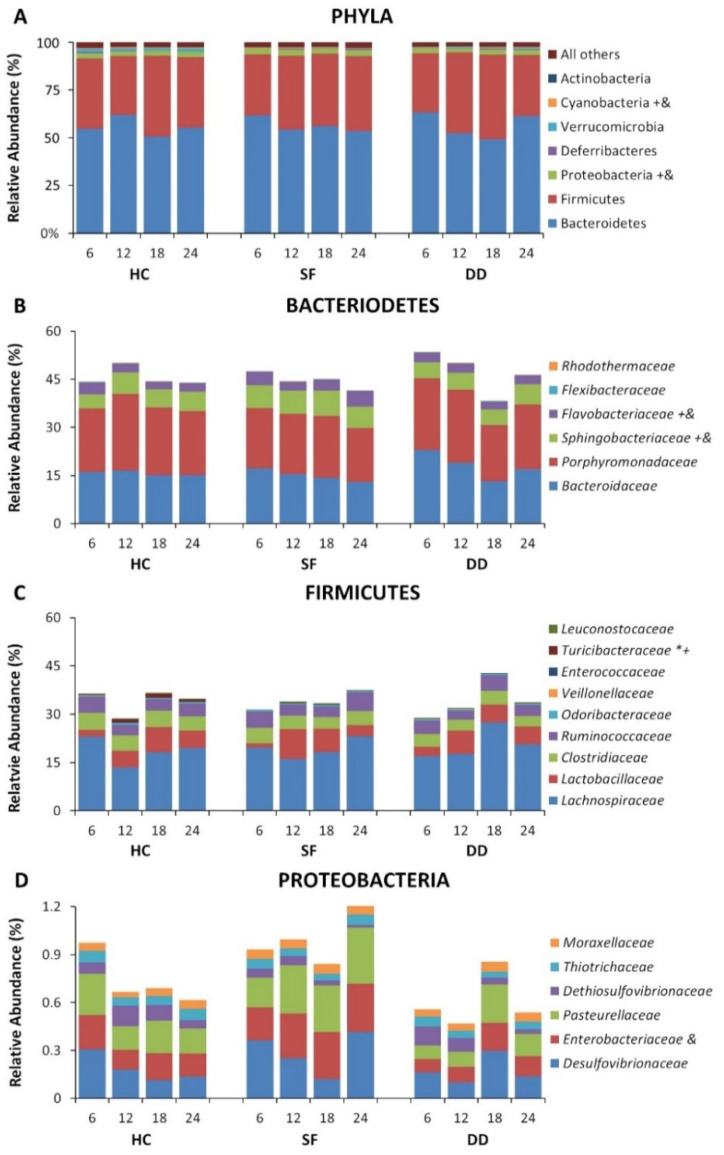
Relative abundance of selected microbiota after Recovery for the home cage control (HC), dark disturbed (DD) and sleep fragmented (SF) experimental groups plotted at 6 h sampling intervals beginning at light onset. (**A**) Relative abundance at the phylum level. (**B**) Relative abundance at the family level for Bacteroidetes. (**C**) Relative abundance at the family level for Firmicutes. (**D**) Relative abundance at the family level for Proteobacteria. Significance is indicated beside the classification name: *, *p* < 0.05, SF compared to control; +, *p* < 0.05 DD compared to control; &, *p* < 0.05 DD compared to Light SS. All Pairwise Multiple Comparison Procedures performed using the Holm–Sidak method. Data at the phylum level are shown as a percentage of all identified phyla. Data at the family level are shown as a percentage of the total identified sequences for the families shown.

Significant alterations were found for major phyla including Bacteriodes, Firmicutes, and Proteobacteria as well as in less prominent phyla. Differences in relative abundance at the phylum level for Bacteroidetes, Firmicutes, and Proteobacteria, and for selected families for the first 10 days are shown in Figure 2. Some microbiota continued to show differences across groups at recovery 10 days after SF had been discontinued, though many of the differences had disappeared (Figure 3). Overall group differences also varied from the experimental period where mice received SF to the recovery period after SF had ended (e.g., Flavobacteriaceae (Experimental: DD > SF; DD > HC, *p* < 0.05; Recovery: HC > DD, *p* < 0.05); Turicibacteraceae (Experimental: DD > SF; DD > HC, *p* < 0.05; Recovery: HC > DD, HC > SF, *p* < 0.05); Coriobacteriaceae (Experimental: HC > DD, HC > SF, *p* < 0.05; Recovery: DD > HC; DD > SF, *p* < 0.05)). These data demonstrate that both light and DD can significantly perturb the microbiome, and that effects can persist after SF has ended. Interestingly, most of the persisting effects were found in mice that had been subjected to DD.

### 3.2. Light and DD Produce Different Effects on Mucosal and Central Immune System

**Figure 4:**
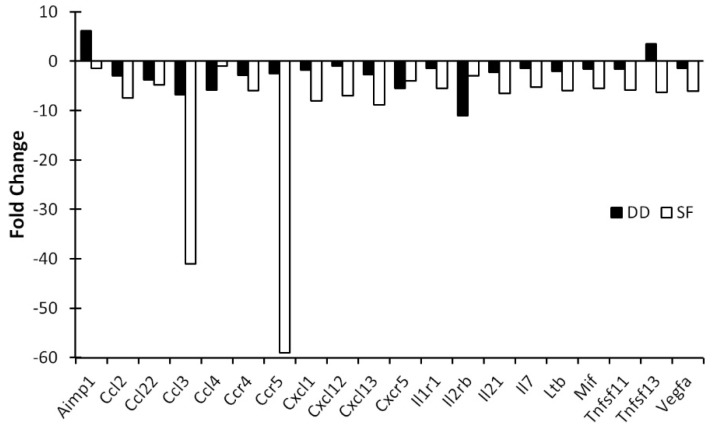
Ten days of SF markedly suppresses MLN cytokine gene expression. MLNs were pooled (4–5 mice/group), RNA isolated and cytokine profiles determined by qPCR array. Data are mean fold change relative to control (non-SF) mice and are plotted for chemokines and cytokines that showed a 5-fold or greater change compared to controls. SF: sleep fragmented; DD: dark disturbed.

To assay potential effects on mucosal immunity, we examined chemokine/cytokine expression in the MLNs which are the primary route for bacterial translocation from the gut, and the first site to show evidence of translocation [19,20] (observing intestinal bacteria in the normally sterile MLN is considered direct evidence of bacterial translocation) [21,22,23,24]. The effects on mucosal immune function were more pronounced for SF. For example, a five-fold or greater reduction in gene expression was observed for 17 genes in SF mice, but for only four genes in DD mice (the multi-function cytokine Aimp1 was increased in DD mice). Of these, notably, CC chemokine receptor 5 (CCR5), which was highly suppressed in the MLNs after SF (Figure 4), is involved in the chemotaxis of leucocytes to inflammation sites [25] and it plays important role in the recruitment of macrophages, T cells, and monocytes in inflammation [26]. SF also more strongly inhibited Ccl3 (but not Ccl4 and Ccl5) expression, chemokines that signal through CCR5 [27,28,29]. Maraviroc, an antagonist for CCR5 used for treating HIV, selectively alters the relative abundance of gut bacteria in mice fed a high fat diet [30]. Cxcl13 was also highly suppressed by SF. This chemokine plays a significant role in the transformation of cryptopatches, produced by microbiota in the lumen, into isolated lymphoid follicles (ILFs) [31]. ILFs act as initiation sites for immunoglobulin A (IgA) responses, which in turn are important for enabling the mucosal immune system to protect against pathogens [31] and for the restoration of homeostasis in intestinal microbiota [32,33,34]. These factors suggest that the suppression of CCR5 and Cxcl13 in the MLNs may be relevant for mediating the effects of SF on the host–microbiota interface. 

**Figure 5:**
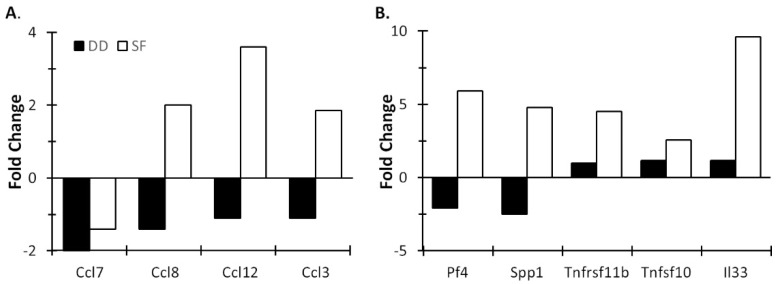
Differential cytokine gene activation in the cortex by SF and DD. Mice were subjected to 10 days of disturbance either during the light or dark period. Mice were then euthanized and cerebral cortex chemokine (**A**) and cytokine profiles (**B**) determined. Brain tissue for each group was pooled (4–5 mice/group), RNA isolated and cytokine profiles determined by qPCR array. Values represent changes in RNA levels relative to undisturbed mice. SF: sleep fragmented; DD: dark disturbed.

**Figure 6:**
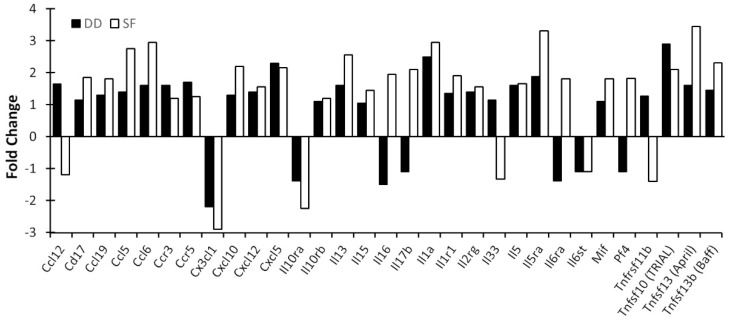
Cytokine gene activation in the mPFC by SF and DD. Mice were subjected to 10 days of disturbance either during the light or dark period. Mice were then euthanized and mPFC chemokine and proinflammatory cytokine profiles determined. Brain tissue for each group was pooled (4–5 mice/group), RNA isolated and cytokine profiles determined by qPCR array. Values represent changes in RNA levels relative to undisturbed mice. SF: sleep fragmented; DD: dark disturbed.

Figure 5 presents data showing the effects of SF and DD on chemokine/cytokine expression in the cerebral cortex. Similar to gene expression profiles seen in the MLNs, SF inhibited expression of the majority of chemokine and proinflammatory cytokine genes (data not shown). However, in contrast with profiles seen in MLNs, SF induced in the cortex expression of a subset of chemokine genes (Figure 5A) as well as cytokine ligands and their receptors (Figure 5B). By comparison, DD suppressed expression of these same genes. Noteworthy among these was Ccl12/MCP-5, a structural and functional homologue of human Ccl2/MCP-1 [35] that is increased in obstructive sleep apnea syndrome [36,37], a sleep disorder characterized by SF. It plays a central role in the inflammatory process by forming a chemoattractant gradient that attracts blood-borne inflammatory cells (neutrophils, monocytes and macrophages) to transmigrate across the blood-brain barrier into the brain [38]. It is thought to coordinate cell movements during early immune response to pathogens and insults (e.g., hypoxia and ischemia) [38]. Another notable induction was the cortical alarmin, interleukin-33 (IL-33), the most potently induced cytokine gene in SF mice. IL-33, a member of the IL-1 family of proinflammatory cytokines [39,40], is released following CNS injury. It acts on astrocytes and microglia to release chemokines that recruit monocytes that promote expression of reparatory M2 macrophage genes, and appears to be important for CNS recovery after injury [41]. IL-33 was much less altered in DD mice compared to controls. SF and DD also differentially influenced the expression of several chemokines and cytokines in the medial prefrontal cortex (mPFC) (Figure 6). These data raise the possibility that chronic SF may lead to persistent, low-grade brain inflammation, as shown for repeated social dominance, a psychological stressor [42], at the same time that mucosal immunity is suppressed. 

## 4. Discussion

Our data indicate that SF can significantly alter gut microbiota and differentially impact mucosal and central immune systems, and that effects can vary depending on whether disturbances were conducted in the light or dark phase. Specifically, SF suppressed important mediators of the mucosal immune system involved in maintaining the gut–microbiota barrier at the same time that a potential neuroinflammatory response was observed in discrete brain regions. Gut microbiota also can influence brain microglia and thereby modulate innate and adaptive immune responses at mucosal surfaces, which are critical for host defense [43]. This suggests that SF, either through effects on gut microbiota and/or other pathways, has the potential to modulate the immune system in ways that could disturb the normal barrier between the gut and resident microbiota. This potential impact of SF is also suggested by the fact that prolonged sleep deprivation can compromise the barrier separating gut microbiota and host and allow bacterial translocation [12,13].

Reciprocal influences of sleep and the immune system have long been recognized [13,44,45,46,47], and sleep disturbance has significant implications for the risk of inflammatory disease [48]. In general, sleep disturbances produce significant elevations in inflammatory markers [49,50,51] and sleep loss is associated with increased circulatory proinflammatory cytokines (IL-1β, IL-6, IL-17A, TNF-α) and C-reactive protein [52]. However, the interrelationships between sleep duration and inflammation are complex and can vary depending on the measure of sleep and the components of the inflammatory response that are examined [53].

Four weeks of SF in the light period has been reported to increase food intake, increase growth in Lachnospiraceae and Ruminococcaceae and decrease Lactobacillaceae families, produce systemic and visceral white adipose tissue inflammation, and to alter insulin sensitivity [16]. In our studies, we conducted an identical 2 min sweep protocol during the dark period to control for stimulation and for potential effects of induced activity. Note that the dark period manipulation may also produce some disturbances in sleep as mice also exhibit sleep during the dark period. However, the value of these two conditions is illustrated by our data which show that both light and dark manipulations produce changes in gut microbiota whereas the impact on immune responses is much more pronounced for SF.

The health impact of disturbed and deficient sleep is significant, as more than 50% of all people in the United States report difficulties with sleep or insufficient sleep at various times, whereas 50 to 70 million report having chronic sleep problems [54]. This disturbance can arise from causes including poor sleep schedules, sleep apnea, stress, and shift work. The clinical implications are profound having been linked with increased obesity, metabolic risk, coronary heart disease and stroke, altered immunity, cancers, and other common diseases [55]. Given the linkage of disturbed sleep and disease, it is not surprising that sleep is integrally related to immune system function. Sleep disturbances alter immune function and immune challenge alters sleep [44]. The reciprocal influences of sleep and the immune system have led to the suggestion that sleep is a component of the acute phase response to infection [45] and that it functions in host defense [46]. Indeed, the adverse effects of prolonged sleep deprivation are linked to host defense failure [13]. It is now becoming widely recognized that sleep loss renders an organism more susceptible to pathogens [45,47] and that during infection, animals that have robust sleep responses have better prospects for survival than those that do not [46]. For instance, rabbits subjected to microbial challenge (Escherichia coli, Staphylococcus aureus, Candida albicans) have a greater chance of survival if they exhibit robust increases in non-rapid eye movement (NREM) sleep and slow wave EEG activity during NREM compared to animals that show extended periods of NREM suppression [46].

The human gastrointestinal (GI) tract alone harbors over 1 × 10^13^ different microbes, a number that exceeds the number of human eukaryotic cells [56]. Recognizably, the intestinal microbiome plays a major influence human health and disease [57]. The exact role(s) these organisms play is not well understood, but evolving evidence indicates that there are interactions among the gut microbiota, the immune system, and the brain (M–G–B axis) that are critical mediators of health and disease [58]. Studies in rodents suggest that gut microbiota can modulate brain development, neurotransmitter systems, signaling pathways, synaptic related proteins, and behavior (reviewed in [1]) via immune, neural, and/or enteroendocrine mechanisms as well as by soluble factors and/or metabolites produced by bacteria [59]. Alterations in the biome balance, known as dysbiosis, contribute to immune dysregulation in the host. Mechanistically this is likely by precipitating a “proinflammatory state” via induction in changes of molecular trafficking, resulting in altered transcription cascades. Sleep disturbance-related dysbiosis upsets a key balance in the interaction between gut flora and the native immune system [60,61]. There are consequent related implications for immune cell activity and gut permeability [62].

Gut microbiota have also been implicated in health-related issues ranging from obesity [58,63,64] to mood and anxiety disorders [1,58,65,66] amongst other disorders. The gut microbiota may also interact with the stress system with implications for both development and adult responsivity to stress [2,67].

The relationship between gut microbiota and the brain is reported to be bi-directional. For example, while gut microbiota may modulate brain function, the brain may alter gut microbiota by changing gastrointestinal motility and intestinal permeability [1,2]. Proper functioning of the M–G–B requires the coordination of complex processes that provide a barrier (provided by the gut mucosal immune system) between tissues of the host organism and the microbes that live within the GI tract [68,69]. Maintaining this barrier enables the host organism to receive the benefits provided by the microbiota including promoting nutrient supply, preventing pathogen colonization, and shaping and maintaining normal mucosa immunity [70]. Failure of the barrier can result in bacterial translocation, defined as the invasion of live microbiota and/or release of their toxins into the MLNs and/or other organs outside the intestines [19]. Though a certain amount of bacterial translocation occurs in healthy humans and animals [19], excessive amounts can lead to the increased risk of sepsis, dysbiosis, cancer, and/or autoimmunity [68] and organ dysfunction and failure [70].

A limitation of our study is that we did not record sleep and, thus, we could not directly assess the changes in sleep architecture that were associated with time under SF. However, we specifically chose to conduct this initial study in non-implanted mice because the procedures necessary for recording sleep via cabling has the potential to impact sleep amounts and activity [71] and recording via telemetry in mice generally requires intraperitoneal implants which would have the potential to alter intestine function [72]. Studies incorporating recording sleep will be needed in the future to fully assess the relationship between sleep changes and alterations in the microbiome and immune markers, but they will need to consider the potential effects of the recording methods that are used. Additionally, assessing changes in the microbiome on different SF schedules and durations will be useful. We conducted our study with ten days of SF and effects have been reported for longer periods [16]. By comparison, Zhang et al. did not find strong evidence of dysbiosis following five days of sleep restriction in rats [73], suggesting that shorter periods of sleep disruption may have fewer observable effects. A last limitation is that the data we present on chemokine and cytokine expression in the MLNs and brain are on pooled samples which may not fully and accurately characterize the changes induced by SF. However, these data should provide a guide for issues that need to be considered in assessing the role of sleep in mediating M–G–B interactions, i.e., sleep disruptions can have effects at multiple points along the M–G–B axis that could play roles in the effects of the microbiome on health and function. They also provide suggestions for chemokines and cytokines that may play roles in regulating M–G–B interactions.

Various studies have demonstrated a relationship between sleep disturbances and gut microbiota that appear to have relevance for health [3,4,5,6,61,74,75]. However, the effects of sleep disruption are complex as it also has a significant interaction with the immune system (e.g., [7,8,9,10,11]) that would have the potential to alter the effects of the gut microbiota, possibly by impact on the immune processes that maintain the barrier between host tissue and the microbiota and/or functioning of the central nervous system. An extreme example of this is found in animals experiencing prolonged, experimental sleep deprivation. Sustained sleep deprivation promotes bacterial translocation [12], and death that can occur after prolonged sleep deprivation in animals has been attributed to bloodstream infection by opportunistic anaerobic bacteria that normally are contained within the gut [13]. Bloodstream infection is also a leading cause of death in critically ill patients [76]. Thus, while experimental prolonged sleep deprivation does not fully model sleep disturbances common in modern life, the recognized effects of sleep deficiency and increasing recognition of the role that the gut microbiota play in mediating immune function suggest that delineating the interactions among disturbed sleep, the immune system and gut microbiota will be critical for understanding their role in health and disease. Mitigation strategies directed at optimizing sleep functionality to maintain health or direct disease-related interventions are potential translational opportunities.

## Data Availability

Experimental data available upon request.
